# Clinical characteristics of inpatients with coronavirus disease 2019 (COVID-19) in Sichuan province

**DOI:** 10.1186/s12879-021-05825-1

**Published:** 2021-02-08

**Authors:** Wen Wang, Lei Chen, Qiao He, Mingqi Wang, Mei Liu, Taibing Deng, Xiaoju Deng, Jianrong Yang, Ou Jiang, Rongmei Li, Bo Long, Gang Mai, Wenhui Huan, Wenquan Li, Xin Jiang, Zeqiang Wen, Yongjun Chen, Wanzhi Fu, Zhiling Long, Fanxin Zeng, Yan Chen, Yihua Du, Juan Tang, Xin Sun, Weimin Li

**Affiliations:** 1 Chinese Evidence-based Medicine Center and Cochrane China Center, West China Hospital, Sichuan University, Chengdu, 610041 Sichuan China; 2 Department of Clinical Research Management, West China Hospital, Sichuan University, Chengdu, 610041 China; 3 Department of Respiratory Disease, Guang’An Hospital, Guangan, 638001 China; 4 Department of Oncology, The People’s Hospital of Yuechi, Yuechi, 638300 China; 5 Department of Infectious Disease, Ganzi Hospital of West China Hospital, Sichuan University, Ganzi, 626000 China; 6 Cancer Center of the Second People’s Hospital of Neijiang, Neijiang, 641100 China; 7 Department of Operation Management, West China Hospital, Sichuan University, Chengdu, 610041 China; 8 Office of the Director, Jintang County First People’s Hospital, Chengdu, 610400 China; 9 Department of Infectious Disease, Sichuan Mianyang 404 Hospital, Mianyang, 621000 China; 10 Department of Hepatopancreatobiliary Surgery, People’s Hospital of Deyang, Deyang, 618000 China; 11 Department of Cardiology, Wusheng County People’s Hospital, Guang’an, 638400 China; 12 Department of Infectious Disease, The Second People’s Hospital of Yibin, West China Yibin Hospital, Sichuan University, Yibin, 644000 China; 13 Mianzhu People’s Hospital, Mianzhu, 618200 China; 14 Department of Thoracic Surgery, The First People’s Hospital of Ziyang, Ziyang, 641300 China; 15 Department of Gastroenterology, Suining Central Hospital, Suining, 629000 China; 16 Department of Infectious Disease, The People’s Hospital of Jianyang, Jianyang, 641400 China; 17 Department of Infectious Disease, Ya’An People’s Hospital, Ya’An, 625000 China; 18 Department of Clinical Research Center, Dazhou Central Hospital, Dazhou, 635000 China; 19 Department of Respiratory and Critical Care Medicine, Nanchong Central Hospital, Nanchong, 637000 China; 20 Department of Pediatric Surgery, The Affiliated Hospital of Southwest Medical University, Luzhou, 646000 China; 21 Department of Infectious Disease, Zigong First People’s Hospital, Zigong, 643000 China; 22 Department of Respiratory and Critical Care Medicine, West China Hospital, Sichuan University, Chengdu, 610041 China

**Keywords:** Novel coronavirus, COVID-19, Clinical characteristics

## Abstract

**Background:**

The outbreak of COVID-19 has resulted in serious concerns in China and abroad. To investigate clinical features of confirmed and suspected patients with COVID-19 in west China, and to examine differences between severe versus non-severe patients.

**Methods:**

Patients admitted for COVID-19 between January 21 and February 11 from fifteen hospitals in Sichuan Province, China were included. Experienced clinicians trained with methods abstracted data from medical records using pre-defined, pilot-tested forms. Clinical characteristics between severe and non-severe patients were compared.

**Results:**

Of the 169 patients included, 147 were laboratory-confirmed, 22 were suspected. For confirmed cases, the most common symptoms from onset to admission were cough (70·7%), fever (70·5%) and sputum (33·3%), and the most common chest CT patterns were patchy or stripes shadowing (78·0%); throughout the course of disease, 19·0% had no fever, and 12·4% had no radiologic abnormality; twelve (8·2%) received mechanical ventilation, four (2·7%) were transferred to ICU, and no death occurred. Compared to non-severe cases, severe ones were more likely to have underlying comorbidities (62·5% vs 26·2%, *P* = 0·001), to present with cough (92·0% vs 66·4%, *P* = 0·02), sputum (60·0% vs 27·9%, *P* = 0·004) and shortness of breath (40·0% vs 8·2%, *P* <  0·0001), and to have more frequent lymphopenia (79·2% vs 43·7%, *P* = 0·003) and eosinopenia (84·2% vs 57·0%, *P* = 0·046).

**Conclusions:**

The symptoms of patients in west China were relatively mild, and an appreciable proportion of infected cases had no fever, warranting special attention.

**Supplementary Information:**

The online version contains supplementary material available at 10.1186/s12879-021-05825-1.

## Background

In December 2019, a cluster of pneumonia cases of unknown etiology occurred in Wuhan, and the first wave of patients all reported exposure history to the Huanan Seafood Wholesale Market [1, 2]. The novel coronavirus was subsequently identified as the origin of the disease (named as SARS-CoV-2), which can cause severe pneumonia and deaths [1, 3]. The outbreak of COVID-19 has resulted in serious concerns in China and abroad [4–9]. By February 26, 2020, a total of 78,497 cases were confirmed by the National Health Commission (NHC) of China [10], and 2918 cases were identified in 37 countries, including Republic of Korea, Japan, and Italy [11, 12].

Despite numerous efforts to investigate the coronavirus and associated diseases, available evidence is still largely inadequate. Currently, only a small number of studies investigated clinical characteristics of the coronavirus infection [3, 13–16]. The first study, a retrospective analysis of 41 laboratory-confirmed cases in Wuhan, found that the coronavirus caused severe respiratory illness similar to SARS; among those, 30% had Intensive Care units (ICU) admissions and 15% were dead [3]. The second retrospective single-center study of 99 confirmed cases in Wuhan reported similar results, with a mortality rate of 11% [15]. However, both studies focused on patients in Wuhan. The epidemiological and clinical features of these patients may differ from those in other places. In addition, due to limited healthcare and human resources to respond the great outbreak in Wuhan, particularly at the early stage, patient outcomes may be altered. Indeed, these two studies reported mortality rates significantly higher than the estimated rate of 2% by the WHO and NHC of China [10, 11].

Three other studies reported patient characteristics and outcomes outside of Wuhan [13, 17, 18]. One included 13 patients from three hospitals in Beijing and suggested that most patients were healthy adults without underlying diseases [13]. A multi-center study, involving 62 COVID-19 cases in Zhejiang province, found that the patient symptoms and prognoses were relatively mild [17]. A most recently published study included 1099 patients both in and outside Wuhan, and suggested a lower case fatality rate than studies exclusively enrolling patients from Wuhan [18]. Two of three studies, however, had relatively small sample sizes and were conducted at the early stage of the disease outbreak, at which time most of the patients (90–100%) either resided or had short-term stays in Wuhan prior to the disease onset [13, 17].

Additionally, no studies specifically reported the situation from west China, where socioeconomic conditions and healthcare facilities are less developed than east regions of China. Therefore, we conducted a study to examine clinical features of infected and suspected patients in Sichuan, a province with the largest population (80 million) in west China.

## Methods

### Study design

We conducted a multi-center retrospective study to investigate the clinical characteristics and outcomes of inpatients with confirmed and suspected COVID-19 in Sichuan province. Sichuan locates in West China and has about 80 million populations. It governs 18 municipal cities and 3 autonomous regions with Yi, Qiang and Tibetan minorities. The first case in Sichuan was confirmed on January 21 [19]. We collected medical records of patients who were admitted to 15 hospitals for COVID-19 from January 21 to February 11. These 15 hospitals covered ten cities and one autonomous region (i.e., Ganzi Tibetan autonomous region), and has treated more than one third patients with COVID-19 in Sichuan. To ensure effective implementation of the study, we developed a multidisciplinary research team, including experts in respiratory medicine and intensive care medicine, epidemiologists, statisticians, and informatics. The Institutional Review Board of West China Hospital approved the study on 10 February 2020 (WCH2020–129), and waived patient consent.

### Case definitions

Confirmed and suspected patients were diagnosed according to the New Coronavirus Pneumonia Prevention and Control Program issued by the National Health Commission of China [20]. Confirmed cases were defined as patients who had a positive result of high-throughput sequencing or real-time reverse-transcriptase polymerase-chain-reaction (RT-PCR) assay for respiratory tract or blood specimens. Suspected cases were identified based on exposure history and clinical features. Patients who resided or traveled to Wuhan, or had close contact with confirmed cases or patients with fever or respiratory symptoms within 14 days were considered as individuals with exposure risk.

For all suspected patients, nucleic acid detections of COVID-19 were performed by the local Center for Disease Control and Prevention, consistent with the WHO protocol [21]. All provinces in China adopted the uniform laboratory testing procedures since January 24. The sequences were detected as following: for Open reading frame 1 ab fragment, the forward primer was 5′-CCCTGTGGGTTTTACACTTAA-3′, reverse primer sequence was 5′-ACGATTGTGCATCAGCTGA-3′, and probe was 5′-FAM-CCGTCTGCGGTATGTGGAAAGGTTATGG-BHQ1–3′; for the N region of the viral sequence, the forward primer was 5′-GGGGAACTTCTCCTGCTAGAAT-3′, reverse primer was 5′-CAGACATTTTGCTCTCAAGCTG-3′, and the probe was5′-FAM-TTGCTGCTGCTTGACAGATT-TAMRA-3’ [18].

Severe COVID-19 was defined in patients meeting any of the following criteria: presence of respiratory distress with an oxygen saturation of blood ≤93%; or oxygenation index ≤300 mmHg. Patients who required care at intensive care unit or mechanical ventilation, or developed shock were defined as critically severe cases [20].

### Data sources and collection

Medical records of patients with confirmed and suspected COVID-19 and those excluded from the infection were photocopied and sent to the data coordination center at West China hospital in Chengdu, Sichuan. A team of experienced respiratory clinicians then reviewed and abstracted data according to a pre-defined, pilot-tested questionnaire, modified from the WHO Case Report Form (CRF). The data coordination center conducted training on data abstractors, and consensus was achieved regarding the rules of data abstraction. The data abstractors collected data by using the Epi-Data software, version 3·1 (EpiData Association), and all abstracted data were checked by a second abstractor.

The CRF included information regarding demographic characteristic (e.g., gender, age), exposure history (e.g., Wuhan exposure and special occupational exposure), symptoms, or signs (e.g., constitutional, respiratory, gastrointestinal symptoms), laboratory and radiologic findings (e.g., routine blood tests, serum creatinine, transaminases, chest X-ray, or computed tomography (CT)), co-morbidities (e.g. diabetes, hypertension, chronic obstructive pulmonary disease (COPD), cerebrovascular disease (CVD)), complications (e.g., acute respiratory distress syndrome (ARDS), shock, or sepsis), treatment pattern (e.g., antiviral, antimicrobial, or supportive treatment), and outcomes (death and admission to ICU). Information regarding symptoms and signs before and after admission was collected separately. We recorded the first laboratory and radiologic findings after admission and the laboratory tests with highest and lowest values during hospitalization.

### Statistical analysis

We summarized clinical features, radiographic and laboratory findings, and treatment patterns for confirmed and suspected patients. Continuous data were summarized as the means and standard deviations or median and interquartile range (IQR). Categorical data were expressed as numbers and percentages.

We compared clinical features between severe and non-severe cases. We applied Wilcoxon rank-sum tests for continuous variables, and used chi-square tests or Fisher’s exact tests for categorical variables. To evaluate potential variables associated with severe cases, we further conducted univariate logistic analysis. Variables related to demographic characteristics, comorbidities, laboratory and radiologic findings were included into model. These analyses were performed using R 3·6·1. All significance tests were two-sided, and a *P* value < 0·050 was used for statistical significance.

## Results

A total of 169 patients were eligible for inclusion. Of these, 147 were laboratory-confirmed cases, including 122 non-severe and 25 severe cases; 22 were suspected cases. Ten were Tibetan ethnic and four were children younger than 6, with the youngest patient aged 2 months. Among included patients, no pregnant women were identified.

### Clinical features of confirmed patients

For confirmed cases, the median age was 44 (IQR, 33–50) years; 57 (38·8%) cases were females; 82 (61·2%) either resided or ever traveled to Wuhan, 23 (19·3%) were infected by imported cases, and two (1·5%) were healthcare workers (Table 1). The most common symptoms of confirmed cases from onset to admission were cough (70·7%), fever (70·5%), and sputum (33·3%), while fatigue (21·8%) and diarrhea (10·2%) were less frequent (Table 2). Almost one-fifth (19·0%) of confirmed patients developed no fever, and 12·2% had no respiratory symptoms throughout the course of disease.

**Table 1:** Basic Characteristics of included patients

	Diagnosis		Disease severity		
	Suspected (***n*** = 22)	Laboratory-confirmed (***n*** = 147)	Non-severe (***n*** = 122)	Severe (***n*** = 25)	***P*** value^#^
**Age, median (IQR), yrs**	51 (34–56)	44 (33–50)	43 (31–49)	50 (43–64)	0.005
**Age groups, No./total (%)**					
< 15 yrs	1/22 (4.6)	1/147 (0.7)	1/122 (0.8)	0/25 (0.0)	0.29^*^
15–44 yrs	8/22 (36.4)	76/147 (51.7)	67/122 (54.9)	9/25 (36.0)	··
45–64 yrs	10/22 (45.4)	54/147 (36·7)	42/122 (34·4)	12/25 (48·0)	··
≥ 65 yrs	3/22 (13·6)	16/147 (10·9)	12/122 (9·8)	4/25 (16·0)	··
**Female sex, No./total (%)**	7/22 (31·8)	57/147 (38·8)	51/122 (41·8)	6/25 (24·0)	0·096
**Ethnic, No./total (%)**	··	··	··	··	0·79
Tibetan	0/22 (0·0)	10/147 (6·8)	8/122 (6·6)	2/25 (8·0)	··
Non-Tibetan	22/22 (100·0)	137/147 (93·2)	114/122 (93·4)	23/25 (92·0)	··
**Exposure history within 14 days, No./total (%)**					
Local residents of Wuhan or recently been to Wuhan	4/13 (30·8)	82/134 (61·2)	66/110 (60·0)	16/24 (66·7)	0·54
Non local: contacted with people from Wuhan	4/12 (33·3)	23/119 (19·3)	21/99 (21·2)	2/20 (10·0)	0·42
Health-care workers	0/14 (0·0)	2/132 (1·5)	2/111 (1·8)	0/21 (0·0)	> 0·99^*^
**Comorbidities, No./total (%)**	6/21 (28·6)	47/146 (32·2)	32/122 (26·2)	15/24 (62·5)	0·0010
Pulmonary diseases	2/21 (9·5)	11/146 (7·5)	5/122 (4·1)	6/24 (25)	0·0020
Chronic obstructive pulmonary diseases	2/21 (9·5)	6/146 (4·1)	3/122 (2·5)	3/24 (12·5)	0·056^*^
Asthma	1/21 (4·8)	1/146 (0·7)	0/122 (0·0)	1/24 (4·2)	0·16^*^
Lung tumor	0/21 (0·0)	1/146 (0·7)	1/122 (0·8)	0/24 (0·0)	> 0·99^*^
Tuberculosis	0/21 (0·0)	3/146 (2·1)	1/122 (0·8)	2/24 (8·3)	0·070^*^
Other comorbidities	6/21 (28·6)	43/146 (29·5)	31/122 (25·4)	12/24 (50·0)	0·030
Hypertension	3/21 (14·3)	19/146 (13·0)	11/122 (9·0)	8/24 (33·3)	0·0040
Diabetes	2/21 (9·5)	10/146 (6·8)	6/122 (4·9)	4/24 (16·7)	0·10
Heart and cardiovascular diseases	2/21 (9·5)	9/146 (6·2)	5/122 (4·1)	4/24 (16·7)	0·061
Chronic kidney diseases	1/21 (4·8)	4/146 (2·7)	2/122 (1·6)	2/24 (8·3)	0·13^*^
Chronic liver diseases	1/21 (4·8)	5/146 (3·4)	4/122 (3·3)	1/24 (4·2)	> 0·99^*^

Abbreviations: *IQR* Interquartile range

^*^ The *P*-value was derived from Fisher’s exact test, two-sided

^#^ *P*-value for the comparison between severe cases versus non- severe infected patients

**Table 2:** Symptoms and Signs of included patients

	Diagnosis		Disease severity		
	Suspected (***n*** = 22)	Laboratory-confirmed (***n*** = 147)	Non-severe (***n*** = 122)	Severe (***n*** = 25)	***P*** value^#^
**Symptoms**					
**Fever from onset to admission, No./total (%)**	16/21 (76·2)	98/139 (70·5)	78/116 (67·2)	20/23 (87·0)	0·10
**Highest temperature from onset to admission, median (IQR), °C**	38·1 (37·8–38·5)	38·0 (37·4–38·5)	37·8 (37·2–38·5)	38·5 (38–38·8)	0·0040
< 37·5	6/19 (31·6)	32/101 (31·7)	30/83 (36·1)	2/18 (11·1)	0·076^*^
37·5–38·0	4/19 (21·1)	32/101 (31·7)	25/83 (30·1)	7/18 (38·9)	··
38·1–39·0	8/19 (42·1)	33/101 (32·7)	26/83 (31·3)	7/18 (38·9)	··
> 39·0	1/19 (5·3)	4/101 (4·0)	2/83 (2·4)	2/18 (11·1)	··
**Fever throughout the course of disease, No./total (%)**	18/22 (81·8)	119/147 (81·0)	96/122 (78·7)	23/25 (92·0)	0·21
**Highest temperature throughout the course of disease, median IQR), °C**	38·3 (37·7–38·5)	38·0 (37·4–38·7)	38·0 (37·3–38·5)	38·5 (38–38·8)	0·0080
< 37·5	5/20 (25·0)	34/131 (26·0)	32/108 (29·6)	2/23 (8·7)	0·025^*^
37·5–38·0	2/20 (20·0)	32/131 (24·4)	27/108 (25·0)	5/23 (21·7)	··
38·1–39·0	11/20 (55·0)	57/131 (43·5)	45/108 (41·7)	12/23 (52·2)	··
> 39·0	2/20 (20·0)	8/131 (6·1)	4/108 (3·7)	4/23 (17·4)	··
**Respiratory symptoms from onset to admission, No./total (%)**	20/22 (90·9)	118/147 (80·3)	95/122 (77·9)	23/25 (92·0)	0·18
Cough	19/22 (86·4)	104/147 (70·7)	81/122 (66·4)	23/25 (92·0)	0·020
Sputum	10/22 (45·5)	49/147 (33·3)	34/122 (27·9)	15/25 (60·0)	0·0040
Shortness of breath	5/22 (22·7)	20/147 (13·6)	10/122 (8·2)	10/25 (40·0)	< 0·0001
**Respiratory symptoms throughout the course of disease, No./total (%)**	21/22 (95·5)	129/147 (87·8)	105/122 (86·1)	24/25 (96·0)	0·30
Cough	20/22 (90·9)	118/147 (80·3)	95/122 (77·9)	23/25 (92·0)	0·18
Sputum production	12/22 (54·5)	76/147 (51·7)	59/122 (48·4)	17/25 (68·0)	0·12
Shortness of breath	7/22 (31·8)	33/147 (22·4)	19/122 (15·6)	14/25 (56·0)	< 0·0001
**Gastrointestinal symptoms from onset to admission, No./total (%)**	1/22 (4·5)	17/147 (11·6)	15/122 (12·3)	2/25 (8·0)	0·79
Diarrhea	1/22 (4·5)	15/147 (10·2)	14/122 (11·5)	1/25 (4·0)	0·45
**Gastrointestinal symptoms throughout the course of disease, No./total (%)**	2/22 (9·1)	54/147 (36·7)	49/122 (40·2)	5/25 (20·0)	0·093
Diarrhea	2/22 (9·1)	43/147 (29·3)	40/122 (32·8)	3/25 (12·0)	0·066
**Other symptoms from onset to admission, No./total (%)**	6/22 (27·3)	59/147 (40·1)	48/122 (39·3)	11/25 (44·0)	0·84
Fatigue	4/22 (18·2)	32/147 (21·8)	27/122 (22·1)	5/25 (20·0)	> 0·99^*^
Chill	1/22 (4·5)	17/147 (11·6)	12/122 (9·8)	5/25 (20·0)	0·27
**Other symptoms throughout the course of disease, No./total (%)**	6/22 (27·3)	74/147 (50·3)	57/122 (46·7)	17/25 (68·0)	0·086
Fatigue	4/22 (18·2)	42/147 (28·6)	34/122 (27·9)	8/25 (32·0)	0·86
Chill	1/22 (4·5)	22/147 (15)	15/122 (12·3)	7/25 (28·0)	0·090
**Signs**					
**Respiratory rate at admission, median (IQR)**	20 (19–21)	20 (20–21)	20 (20–20)	21 (20–22)	0·0020
**SBP at admission, median (IQR), mmHg**	127 (117–135)	126 (116–139)	126 (116–138)	128 (119–139)	0·38
> 140 mmHg, No·/total (%)	4/17 (23·5)	26/129 (20·2)	21/108 (29·4)	5/21 (23·8)	0·87
**DBP at admission, median (IQR), mmHg**	80 (74–86)	79 (72–88)	79 (71–88)	80 (75–86)	0·94
> 90 mmHg, No·/total (%)	3/17 (17·6)	28/129 (21·7)	24/108 (22·2)	4/21 (19·0)	0·97
**Heart rate at admission, median (IQR)**	90 (80–106)	90 (80–103)	90 (80–99)	104 (91–109)	0·0050

Abbreviations: *IQR* Interquartile range, *SBP* Systolic Blood Pressure, *DBP* Diastolic Blood Pressure

^*^ The *P*-value was derived from Fisher’s exact test, two-sided

^#^ P-value for the comparison between severe cases versus non- severe infected patients

**Fig. 1:**
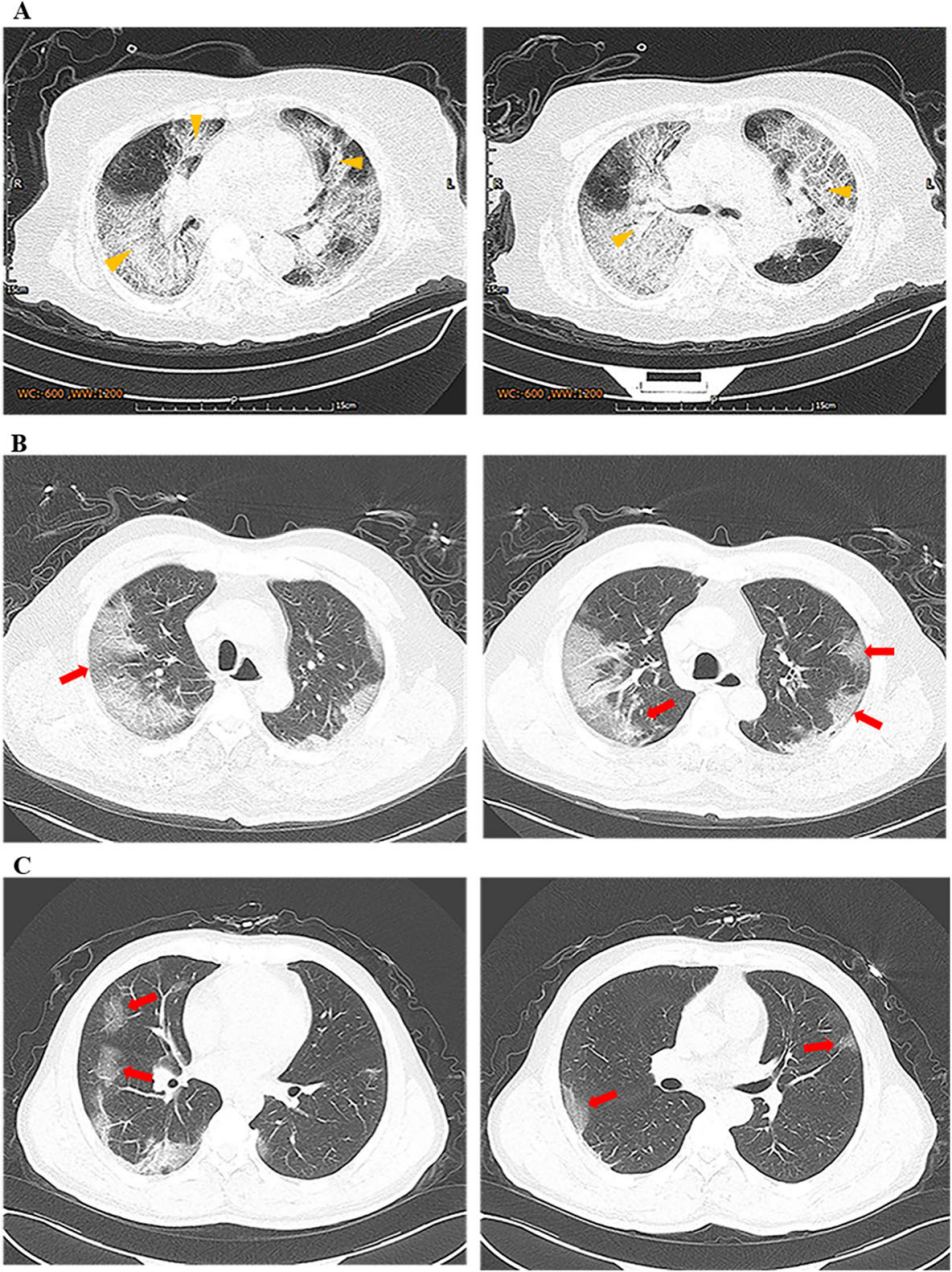
Transverse chest computed tomograms of three patients. **a** Chest CT images showing bilateral diffuse ground glass opacity and subsegmental areas of consolidation on day 4 after symptom onset. **b** Chest CT images showing bilateral multiple ground glass opacity on day 10 after symptom onset. **c** Chest CT images showing scattered ground glass opacity on day 7 after symptom onset. Yellow arrow: consolidation; Red arrow: ground-glass opacities

Among confirmed patients, lymphopenia and eosinopenia was reported in 71 (49·7%) and 81 (60·9%), respectively, 113 (87·6%) had abnormal chest CT findings in the first examination, and 118 (89·4%) had abnormal CT findings during hospitalization (Table 3). The median time from illness onset to abnormalities on CT was 3 days. The most common patterns on chest CT during hospitalization were patchy or stripes shadowing (78·0%), ground-glass opacity (74·2%), and most were bilateral pneumonia (76·5%, Table 3 and Fig. 1).

**Table 3:** Laboratory and radiologic findings of included patients

	Diagnosis		Disease severity		
	Suspected (***n*** = 22)	Laboratory-confirmed (***n*** = 147)	Non-severe (***n*** = 122)	Severe (***n*** = 25)	***P*** value^#^
**Laboratory findings at first test**					
**White blood cell count, median (IQR), × 10**^**9**^**/L**	6·7 (5·0–9·6)	5·0 (4·0–6·1)	4·9 (4·0–6·0)	5·3 (4·5–7·4)	0·054
> 10× 10^9^/L, No·/total (%)	3/13 (23·1)	9/145 (6·2)	4/120 (3·3)	5/25 (20·0)	0·0070
< 4× 10^9^/L, No·/total (%)	2/13 (15·4)	18/145 (12·4)	15/120 (12·5)	3/25 (12·0)	> 0·99
**Lymphocyte count, median (IQR), × 10**^**9**^**/L**	1·00 (0·80–1·20)	1·14 (0·90–1·60)	1·20 (1·00–1·70)	0·80 (0·50–1·00)	< 0·0001
< 1·1× 10^9^/L, No·/total (%)	6/11 (54·5)	71/143 (49·7)	52/119 (43·7)	19/24 (79·2)	0·0030
**Eosinophils count, median (IQR), × 10**^**9**^**/L**	0·02 (0·00–0·05)	0·01 (0·00–0·05)	0·02 (0–0·05)	0·00 (0·00–0·01)	0·0030
< 0·02× 10^9^/L, No·/total (%)	6/11 (54·5)	81/133 (60·9)	65/114 (57·0)	16/19 (84·2)	0·046
**Haemoglobin, median (IQR), g/L**	133 (125–151)	142 (127–156)	144 (128–155)	142 (124–157)	0·87
< 130 g/L, No·/total (%)	6/13 (46·2)	42/143 (29·4)	34/118 (28·8)	8/25 (32·0)	0·94
**International normalised ratio, median (IQR)**	1·05 (0·97–1·08)	1·04 (0·97–1·13)	1·03 (0·97–1·13)	1·04 (1·00–1·11)	0·58
> 1·26, No·/total (%)	0/7 (0·0)	6/125 (4·8)	6/108 (5·6)	0/17 (0·0)	> 0·99^*^
**D-dimer, median (IQR), μg/L**	390 (280–430)	195 (93–403)	175 (90–368)	414 (163–930)	0·025
> 550 μg/L, No·/total (%)	1/5 (20·0)	22/122 (18·0)	16/104 (15·4)	6/18 (33·3)	0·13
**Albumin, median (IQR), g/L**	41·2 (34·7–45·1)	43·7 (40·9–46·1)	44·2 (42·1–46·9)	38·2 (35·3–43·8)	< 0·0001
< 35 g/L, No·/total (%)	3/12 (25·0)	7/136 (5·2)	2/114 (1·6)	5/22 (22·7)	< 0·0001
**Alanine aminotransferase, median (IQR), U/L**	22 (15–25)	26 (17–46)	26 (17–46)	29 (21–41)	0·46
> 50 U/L, No·/total (%)	0/10 (0·0)	26/136 (19·1)	21/116 (18·1)	5/20 (25·0)	0·68
**Aspartate aminotransferase, median (IQR), U/L**	24 (22–30)	26 (20–35)	26 (20–36)	25 (21–34)	0·64
> 40 U/L, No·/total (%)	0/8 (0·0)	18/108 (16·7)	15/91 (16·5)	3/17 (17·6)	> 0·99^*^
**Total bilirubin, median (IQR), μmol/L**	9·7 (6·0–18·0)	10·5 (7·0–16·9)	10·8 (7·0–16·5)	8·7 (7·0–18·3)	0·94
> 28 μmol/L, No·/total (%)	1/12 (8·3)	9/135 (6·7)	7/116 (6·3)	2/19 (10·5)	0·81
**Direct bilirubin, median (IQR), μmol/L**	3·9 (2·8–7·3)	4·0 (2·7–5·8)	3·9 (2·6–5·4)	4·0 (3·0–6·7)	0·30
> 10 μmol/L, No·/total (%)	2/12 (16·7)	9/135 (6·7)	7/116 (6·0)	2/19 (10·5)	0·82
**Serum creatinine, median (IQR), μmol/L**	80·5 (69·3–89·3)	74·3 (59·7–83·5)	72·5 (59·0–83·5)	76·2 (63·8–83·5)	0·57
> 106 μmol/L, No·/total (%)	1/12 (8·3)	5/132 (3·8)	4/112 (3·8)	1/20 (5·0)	0·57^*^
**Troponin T, median (IQR), pg/mL**	3·00 (1·10–47·12)	3·00 (0·03–9·7)	3·00 (0·03–9·15)	6·13 (0·23–13·40)	0·23
> 14 pg/mL, No·/total (%)	3/7 (42·9)	8/80 (10·0)	5/67 (7·5)	3/13 (23·1)	0·23
**Procalcitonin, median (IQR), ng/mL**	0·04 (0·03–0·21)	0·05 (0·03–0·06)	0·05 (0·03–0·06)	0·05 (0·02–0·08)	0·56
> 0·05 ng/mL, No·/total (%)	4/13 (30·8)	45/128 (35·2)	36/107 (33·6)	9/21 (42·9)	0·58
**Erythrocyte sedimentation rate, median (IQR), mm/h**	27 (21–51)	22 (10–36)	21 (10–33)	23 (7–44)	0·77
> 15 mm/h, No·/total (%)	9/11 (81·8)	64/102 (62·7)	54/88 (61·4)	10/14 (71·4)	0·67
**C-reactive protein, median (IQR), mg/L**	7·6 (0·9–31·8)	7·2 (2·0–24·2)	5·7 (1·9–15·6)	31·7 (14·2–54·2)	< 0·0001
**Procalcitonin, median (IQR), ng/mL**	0·04 (0·03–0·21)	0·05 (0·03–0·06)	0·05 (0·03–0·06)	0·05 (0·02–0·08)	0·56
> 0·05 ng/mL, No·/total (%)	4/13 (30·8)	45/128 (35·2)	36/107 (33·6)	9/21 (42·9)	0·58
**Erythrocyte sedimentation rate, median (IQR), mm/h**	27 (21–51)	22 (10–36)	21 (10–33)	23 (7–44)	0·77
> 15 mm/h, No·/total (%)	9/11 (81·8)	64/102 (62·7)	54/88 (61·4)	10/14 (71·4)	0·67
**C-reactive protein, median (IQR), mg/L**	7·6 (0·9–31·8)	7·2 (2·0–24·2)	5·7 (1·9–15·6)	31·7 (14·2–54·2)	< 0·0001
> 5 mg/L, No·/total (%)	6/12 (50·0)	62/122 (50·8)	44/102 (43·1)	18/20 (90·0)	< 0·0001
**CKMB**^**a**^					
Increased, No·/total (%)	2/11 (18·2)	7/91 (7·7)	6/79 (7·6)	1/12 (8·3)	> 0·99^*^
**Gamma-glutamyltran sferase, median (IQR), U/L**	20 (18–34)	30 (17–57)	29 (16–46)	33 (17–73)	0·350
**Lactate dehydrogenase, median (IQR), U/L**	245 (179–276)	187 (167–233)	184 (163–227)	229 (190–400)	0·015
**Potassium, median (IQR), mmol/L**	4·2 (3·8–4·3)	3·8 (3·6–4·1)	3·8 (3·6–4·1)	3·8 (3·4–4·1)	0·86
**Sodium, median (IQR), mmol/L**	140 (136–142)	139 (136–141)	139 (137–141)	137 (135–139)	0·011
**Radiologic findings**					
**Abnormalities at first examination, No./total (%)**	13/14 (92·9)	113/129 (87·6)	94/109 (86·2)	19/20 (95·0)	0·47
Bilateral pneumonia	10/14 (71·4)	87/129 (67·4)	68/109 (62·4)	19/20 (95·0)	0·0090
Unilateral pneumonia	3/14 (21·4)	26/129 (20·2)	26/109 (23·9)	0/20 (0·0)	0·032
Ground-glass opacity	6/14 (42·9)	89/129 (69·0)	73/109 (67·0)	16/20 (80·0)	0·37
Patchy or stripes shadowing	10/14 (71·4)	89/129 (69·0)	75/109 (68·8)	14/20 (70·0)	> 0·99^*^
Parenchymal abnormalities	2/14 (14·3)	21/129 (16·3)	16/109 (14·7)	5/20 (25·0)	0·41
Pleural effusion	0/14 (0·0)	5/129 (3·9)	2/109 (1·8)	3/20 (15·0)	0·026^*^
**Abnormalities during hospitalization, No./total (%)**	13/14 (92·9)	118/132 (89·4)	98/112 (87·5)	20/20 (100·0)	0·20
Bilateral pneumonia	11/14 (78·6)	101/132 (76·5)	81/112 (72·3)	20/20 (100·0)	0·016
Unilateral pneumonia	2/14 (14·3)	17/132 (12·9)	17/112 (15·2)	0/20 (0·0)	0·13
Ground-glass opacity	7/14 (50·0)	98/132 (74·2)	80/112 (71·4)	18/20 (90·0)	0·14
Patchy or stripes shadowing	12/14 (85·7)	103/132 (78·0)	87/112 (77·7)	16/20 (80·0)	> 0·99^*^
Parenchymal abnormalities	2/14 (14·3)	31/132 (23·5)	25/112 (22·3)	6/20 (30·0)	0·65
Pleural effusion	0/14 (0·0)	6/132 (4·5)	3/112 (2·7)	3/20 (15·0)	0·045^*^

Abbreviations: *IQR* Interquartile range, *CKMB* Creatine Kinase-MB

^*^ The *P*-value was derived from Fisher’s exact test, two-sided; ^#^ *P*-value for the comparison between severe cases versus non- severe infected patients; ^a^Classified by different reference range of hospitals

The median time from disease onset to first medical visit was 1 day (IQR, 0–5). Nine (6·2%) confirmed patients developed respiratory failure, and four (2·7%) developed secondary bacterial pneumonia. A total of 144 (98·6%) patients received antiviral therapy, 81 (56·3%) received antibiotics, and 34 (27·0%) received glucocorticoids. Twelve (8·2%) patients required mechanical ventilation, of which 11 (7·5%) received non-invasive ventilation, and two (1·4%) received invasive mechanical ventilation. Four (2·7%) confirmed patients were transferred to intensive care unit, and no patients died (Table 4).

**Table 4:** Complications, treatment pattern and clinical outcomes of included patients

	Diagnosis		Disease severity		
	Suspected (***n*** = 22)	Laboratory-confirmed (***n*** = 147)	Non-severe (***n*** = 122)	Severe (***n*** = 25)	***P*** value^#^
**Complications**					
**Any Complications**	2/19 (10·5)	15/146 (10·3)	3/122 (2·5)	12/24 (50·0)	< 0·0001
Respiratory failure	1/19 (5·3)	9/146 (6·2)	2/122 (1·6)	7/24 (29·2)	< 0·0001
Secondary bacterial pneumonia	0/19 (0·0)	4/146 (2·7)	0/122 (0·0)	4/24 (16·7)	> 0·99^*^
Coagulation disorders	0/19 (0·0)	2/146 (1·4)	0/122 (0·0)	2/24 (8·3)	0·03^*^
Metabolic acidosis	0/19 (0·0)	1/146 (0·7)	0/122 (0·0)	1/24 (4·2)	0·16^*^
Shock	0/19 (0·0)	1/146 (0·7)	0/122 (0·0)	1/24 (4·2)	0·16^*^
Bacteremia or Sepsis	1/19 (5·3)	2/146 (1·4)	0/122 (0·0)	2/24 (8·3)	0·026^*^
Acute lung injury or ARDS	0/19 (0·0)	1/146 (0·7)	0/122 (0·0)	1/24 (4·2)	0·16^*^
Acute renal injury	0/19 (0·0)	0/146 (0·0)	0/122 (0·0)	0/24 (0·0)	··
**Treatment**					
**Antiviral treatment**	11/14 (78·6)	144/146 (98·6)	119/121 (98·3)	25/25 (100·0)	> 0·99^*^
Ribavirin	3/14 (21·4)	14/146 (9·6)	10/121 (8·3)	4/25 (16·0)	0·23
Interferon	8/14 (57·1)	95/146 (65·1)	77/121 (63·6)	18/25 (72·0)	0·43
Lopinavir/Litonavir	4/14 (28·6)	133/146 (91·1)	114/121 (94·2)	19/25 (76·0)	0·0040
Oseltamivir	4/14 (28·6)	22/146 (15·1)	17/121 (14·1)	5/25 (20·0)	0·45
**Antibiotic treatment**	9/16 (56·3)	81/144 (56·3)	59/120 (49·2)	22/24 (91·7)	< 0·0001
**Glucocorticoids**	1/15 (6·7)	34/126 (27·0)	16/101 (15·8)	18/25 (72·0)	< 0·0001
**Intravenous immunoglobulin therapy**	1/22 (4·5)	8/147 (5·4)	4/122 (3·3)	4/25 (16·0)	0·010
**Oxygen therapy**	7/22 (31·8)	59/147 (40·1)	41/122 (33·6)	18/25 (72·0)	< 0·0001
**Mechanical ventilation (MV)**	2/22 (9·1)	12/147 (8·2)	0/122 (0·0)	12/25 (48·0)	< 0·0001
Non-invasive MV	2/22 (9·1)	11/147 (7·5)	0/122 (0·0)	11/25 (44·0)	< 0·0001
Invasive MV	1/22 (4·5)	2/147 (1·4)	0/122 (0·0)	2/25 (8·0)	0·029^*^
**ECMO**	0/22 (0·0)	0/147 (0·0)	0/122 (0·0)	0/25 (0·0)	··
**CRRT**	0/22 (0·0)	1/147 (0·7)	0/122 (0·0)	1/25 (4·0)	0·17^*^
**Clinical outcomes**					
**ICU admission**	0/22 (0·0)	4/147 (2·7)	0/122 (0·0)	4/25 (16·0)	0.00068
**Death**	0/22 (0·0)	0/147 (0·0)	0/122 (0·0)	0/25 (0·0)	··

Values are No./total No. (%)

Abbreviations: *ICU* Intensive Care Units, *ECOM* Extracorporeal Membrane Oxygenation, *CRRT* Continuous Renal Replacement Therapy, *ARDS* Acute Respiratory Distress Syndrome

^*^ The *P*-value was derived from Fisher’s exact test, two-sided

^#^ *P*-value for the comparison between severe cases versus non- severe infected patients

### Clinical features of suspected patients

Among 22 suspected patients, seven (31·8%) were females, and the median age was 51 (IQR, 34–56) years. Four (30·8%) either resided or ever traveled to Wuhan, and four (33·3%) contacted with people from Wuhan. The most common symptoms from onset to admission were cough (86·4%), fever (76·2%), and sputum (45·5%) (Table 2). Lymphopenia and eosinopenia occurred in 6 (54·5%) of suspected patients. Thirteen (92·9%) suspected patients had abnormal CT findings, and the most common patterns were patchy or stripes shadowing (85·7%) during hospitalization (Table 3). One (5·3%) patients developed respiratory failure, and two (9·1%) received non-invasive mechanical ventilation (Table 4).

### Comparisons between severe and non-severe infected patients

The median age of severe and non-severe cases was 50 and 43 years (*P* = 0·73). Compared to non-severe cases, severe ones were more likely to have underlying comorbidities (62·5% vs 26·2%, *P* = 0·0010), including hypertension (33·3% vs 9·0%, *P* = 0·0040) and pulmonary diseases (25·0% vs 4·1%, *P* = 0·0020) (Table 1). The temperature was higher in severe cases both from onset to admission (38·5 °C vs 37·8 °C, *P* = 0·004) and throughout the course of disease (38·5 °C vs 38 °C, *P* = 0·0080). Respiratory symptoms during onset to admission were more commonly presented in severe cases than non-severe ones, including cough (92·0% vs 66·4%, *P* = 0·020), sputum (60·0% vs 27·9%, *P* = 0·0040) and shortness of breath (40·0% vs 8·2%, *P* <  0·0001). The heart rates at admission were higher in severe cases (104 times per minute [IQR, 91–109] vs 90 times per minute [IQR, 80–99], *P* = 0·0050, Table 2). No significant differences were found in gastrointestinal symptoms between the two populations, although appearing higher in non-severe cases both from onset to admission (8·0% vs 12·3%) and throughout the course of disease (20·0% vs 40·2%).

Severe cases had more abnormalities on the first laboratory tests, including lower lymphocyte counts (0·8 × 10^9^/L [IQR, 0·5–1·0]) vs 1·2 × 10^9^/L [IQR, 1·0–1·7], *P* <  0·0001) and eosinophils count (0·00 × 10^9^/L [IQR, 0·00–0·01] vs. 0·02 × 10^9^/L [IQR, 0·00–0·05], *P* = 0·0030), and higher proportion of lymphopenia (79·2% vs 43·7%, *P* = 0·0030) and eosinopenia (84·2% vs 57·0%, *P* = 0·046) (Table 3). Severe patients also had a higher level of D-dimer (414 μmol/L [IQR, 163–930] vs 175 μmol/L [IQR, 90–368], *P* = 0·025) and C-reactive protein (31·7 mg/L [IQR, 14·2–54·2] vs 5·7 mg/L [IQR, 1·9–15·6], *P* <  0·0001). The finding during hospitalization were similar to the first laboratory tests (Supplementary Table 1).

During hospitalization, severe cases were more likely to have bilateral pneumonia (100·0% vs. 72·3%, *P* = 0·016) and pleural effusion (15·0% vs. 2·7%, *P* = 0·045, Table 3), and were more likely to receive antibiotics (91·7% vs 49·2%, *P* <  0·0001), glucocorticoids (72·0% vs 15·8%, *P* <  0·0001), intravenous immunoglobulins (16·0% vs 3·3%, *P* = 0·010) and oxygen therapies (72·0% vs 33·6%, *P* <  0·0001) (Table 4).

### Factors associated with severe cases

Univariate logistic analysis showed that patients with pulmonary diseases (OR 7·80, 2·14–29·72), hypertension (OR 5.05, 1.73–14.48), white blood cell count > 10× 10^9^/L.

(OR 7.38, 1.81–32.10), lymphocyte count< 1·1× 10^9^/L (OR 4·26, 1·67–12·39), bilateral pneumonia (OR 11·46, 2·24–209·65), and pleural effusion (OR 9·44, 1·47–75·78) were more likely to develop into severe cases. Higher temperature (OR 2.52, 1.37–5.08)) and higher heart rate (OR 1.04, 1.01–1.07) were associated with increased risk of developing severe cases (Table 5).

**Table 5:** Factors associated with severe cases with COVID-19

	N (%)	Crude OR (95%CI)	*P* value
Age > 65 yrs	15 (10·2)	1·92 (0·50, 6·24)	0·3
Female sex	57 (38·8)	0·44 (0·15, 1·12)	0·102
Comorbidities			
Pulmonary diseases	11 (7·5)	7·80 (2·14, 29·72)	0·002
Hypertension	19 (13·0)	5·05 (1·73, 14·48)	0·003
Symptoms and Signs			
Highest temperature throughout the course the disease	38 (37·4–38·7)	2·52 (1·37, 5·08)	0·005
Cough throughout the course the disease	118 (80·3)	3·27 (0·89, 21·17)	0·123
Respiratory rate at admission	20 (20–21)	1·07 (0·97, 1·20)	0·165
Heart rate at admission	90 (80–103)	1·04 (1·01, 1·07)	0·013
Laboratory and radiologic findings at admission			
White blood cell count > 10× 10^9^/L	9 (6·2)	7·38 (1·81, 32·10)	0·005
Lymphocyte count< 1·1× 10^9^/L	71 (49·7)	4·26 (1·67, 12·39)	0·003
Eosinophils count< 0·02× 10^9^/L	81 (60·9)	1·56 (0·65, 3·94)	0·329
C-reactive protein	7·2 (2·0–24·2)	1·00 (1·00, 1·02)	0·147
Bilateral pneumonia	87 (67·4)	11·46 (2·24, 209·65)	0·020
Pleural effusion	5 (3·9)	9·44 (1·47, 75·78)	0·018

Abbreviations: *OR* Odds ratio

## Discussion

### Main findings and implications

In this study, we found that most confirmed COVID-19 cases were adults, particularly males. The most common symptoms of confirmed patients were cough, fever and sputum, while gastrointestinal symptoms were less frequent. Presence of lymphopenia and eosinopenia was also frequent. A typical finding of CT scan for COVID-19 was bilateral ground-glass opacity, occurring in two-third of patients. Nearly all patients received antiviral treatments, with lopinavir/litonavir being the most often used.

Generally, the symptoms of patients in Sichuan were relatively mild, and the clinical outcomes were better than those in Wuhan. We found that about 19·0% of confirmed cases did not have fever, and 10·6% had no radiologic abnormality throughout the course of disease. In contrast, the proportion of absence of fever and radiologic abnormality were reported less than 5% in Wuhan [3, 14, 15]. Only two (1·5%) healthcare workers were infected, the proportion of which was much lower than that in Wuhan [14, 16]. Among the 147 confirmed cases in Sichuan, 12 (8·2%) received mechanical ventilations and no death occurred, which contrasted the reported mortality ranging from 4·3% to 15·0% in Wuhan [3, 14, 15, 22]. Similar to our findings, Xu et al also suggested that patient symptoms in Zhejiang province were mild [17]. The differences in the clinical features and outcomes between Wuhan versus other regions may be due to the facts that limited healthcare and human resources were available in Wuhan, particularly at the early stage of the outbreak. Indeed, the median time from disease onset to the first medical visit was 1 day in Sichuan as opposed to 7 days in Wuhan [14].

The COVID-19 had both similar and distinct characteristics in comparison with severe acute respiratory syndrome coronavirus (SARS-CoV) [23]. Although symptoms were similar [3, 15, 16], a fever-free condition was observed in 19% of confirmed cases in our study, much higher than that in SARS-CoV (less than 1%) [24, 25]. SARS-CoV was clearly a more serious condition, with about 17·0% of patients receiving invasive mechanical ventilations and 9·6% died [24]. In contrast, our study showed that 2·7% of COVID-19 patients were transferred to ICU, and 1·4% received invasive mechanical ventilation. Until now, the human has not developed specific antiviral drugs for both coronaviruses.

Our study found that older patients and those with underling comorbidities were more likely to develop severe illness, consistent with other reported findings [3, 14, 15]. We also found that severe cases were more likely to have fever, respiratory symptoms, abnormal laboratory and radiologic findings, and to develop complications including respiratory failure, coagulation disorders, and sepsis than non-severe cases. These are all consistent with published studies [3, 14, 22]. In particular, we found that eosinopenia appeared in 57·0% and 84·2% of non-severe cases and severe cases, suggesting a potential of use for differentiating disease severity. Indeed, eosinopenia has been identified as a good diagnostic marker for severe infections, such as sepsis and bloodstream infection [26, 27]. A study including 177 ICU patients suggested that eosinopenia may be a good diagnostic marker in distinguishing between non-infection and infection with an area under receiver operating characteristic curve of 0·89 [26]. A possible explanation was that eosinophils migrated to the inflammatory site due to chemotactic substances released during acute inflammation [26].

### Strengths and limitations

Our study has several strengths. To our best knowledge, this is the first study investigating clinical characteristics of COVID-19 patients from west China. To ensure representativeness, we collected data from 15 hospitals specifically responsible for treating COVID-19 patients. We also implemented rigorous approaches to collect clinical data to ensure the quality of data.

Meanwhile, our study was a retrospective study, and the data accuracy and completeness were not optimal. Nevertheless, we implemented a strong data collection strategy to minimize potential bias. Secondly, most patients were hospitalized at the time of data collection. Thus, we were unable to investigate outcomes of those infected patients. Thirdly, we included a limited number of patients. As such, we were unable to conduct more sophisticated analyses to control for potential confounding effect. Fourthly, the total number of patients visiting the studied hospitals is not available, and hence the proportions of suspected and confirmed patients with COVID-19 among all hospital visits were unclear.

## Conclusion

In conclusion, the most common symptoms of COVID-19 were cough, fever and sputum, and an appreciable proportion of confirmed cases were absent from fever during the course of the disease. The symptoms of patients in Sichuan province were relatively mild.

## Supplementary Information

**Additional file 1: Supplementary Table 1.**·Laboratory findings of included patients during hospitalization.

## Data Availability

All material and data described in the manuscript are available upon request to the corresponding author of the present article.

## Abbreviations

COVID-19: Novel coronavirus
ICU: Intensive Care Units
RT-PCR: Real-Time Reverse-transcriptase polymerase-chain-reaction
CRF: Case Report Form
CT: Computed Tomography
COPD: Chronic Obstructive Pulmonary Disease
CVD: Cerebrovascular Disease
ARDS: Acute Respiratory Distress Syndrome
IQR: Interquartile Range
SARS-CoV: Severe Acute Respiratory Syndrome Coronavirus
SBP: Systolic Blood Pressure
DBP: Diastolic Blood Pressure
CKMB: Creatine Kinase-MB
ECOM: Extracorporeal Membrane Oxygenation
CRRT: Continuous Renal Replacement Therapy
