# Supplementary material for: Clinical characteristics of inpatients with coronavirus disease 2019 (COVID-19) in Sichuan province
Source: BMC Infect Dis. 2021 Feb 8;21:155. doi: 10.1186/s12879-021-05825-1 (PMC7868861; doi:10.1186/s12879-021-05825-1)
Supplement: Supplementary file 1 — Additional file 1: Supplementary Table 1.·Laboratory findings of included patients during hospitalization. [file 12879_2021_5825_MOESM1_ESM.docx]

**Supplementary Table 1· Laboratory findings of included patients during hospitalization**

|  | **Diagnosis** | |  | **Disease severity** | | |
| --- | --- | --- | --- | --- | --- | --- |
|  | **Suspected**  **(n = 22)** | **Laboratory-confirmed**  **(n = 147)** |  | **Non-severe**  **(n = 122)** | **Severe**  **(n = 25)** | **P value** ^#^ |
| **White blood cell count, median (IQR), × 10⁹/L** | 6·7 (5·0-9·6) | 5·0 (4·0-6·1) |  | 4·9 (4·0-6·0) | 5·3 (4·5-7·4) | 0·054 |
| > 10× 10⁹/L, No./total (%) | 4/13 (30·8) | 24/145 (16·6) |  | 15/120 (12·5) | 9/25 (36·0) | 0·010 |
| < 4× 10⁹/L, No./total (%) | 2/13 (15·4) | 32/145 (22·1) |  | 25/120 (20·8) | 7/25 (28·0) | 0·60 |
| **Lymphocyte count, median (IQR), × 10⁹/L** | 1·00 (0·80-1·20) | 1·14 (0·90-1·60) |  | 1·20 (1·00-1·70) | 0·80 (0·50-1·00) | < 0·0001 |
| < 1·1× 10⁹/L, No./total (%) | 7/11 (63·6) | 82/143 (57·3) |  | 60/119 (50·4) | 22/24 (91·7) | < 0·0001 |
| **Eosinophils count, median (IQR), × 10⁹/L** | 0·02 (0·00-0·05) | 0·01 (0·00-0·05) |  | 0·02 (0·00-0·05) | 0·00 (0·00-0·01) | 0·0030 |
| < 0·02× 10⁹/L, No./total (%) | 6/11 (54·5) | 88/133 (66·2) |  | 71/114 (62·3) | 17/19 (89·5) | 0·040 |
| **Haemoglobin, median (IQR), g/L** | 133 (125-151) | 142 (127-156) |  | 144 (128-155) | 142 (124-157) | 0·87 |
| < 130 g/L, No./total (%) | 6/13 (46·2) | 53/143 (37·1) |  | 43/118 (36·4) | 10/25 (40·0) | 0·92 |
| **International normalised ratio, median (IQR)** | 1·05 (0·97-1·08) | 1·04 (0·97-1·13) |  | 1·03 (0·97-1·13) | 1·04 (1·00-1·11) | 0·58 |
| > 1·26, No·/total (%) | 0/7 (0·0) | 8/125 (6·4) |  | 6/108 (5·6) | 2/17 (11·8) | 0·66 |
| **D-dimer, median (IQR), µg/L** | 390 (280-430) | 195 (93-403) |  | 175 (90-368) | 414 (163-930) | 0·025 |
| > 550µg/L, No./total (%) | 1/5 (20·0) | 26/122 (21·3) |  | 18/104 (17·3) | 8/18 (44·4) | 0·020 |
| **Albumin, median (IQR), g/L** | 41·2 (34·7-45·1) | 43·7 (40·9-46·1) |  | 44·2 (42·1-46·9) | 38·2 (35·3-43·8) | < 0·0001 |
| < 35 g/L, No./total (%) | 4/12 (33·3) | 16/136 (11·8) |  | 6/114 (5·3) | 10/22 (45·5) | < 0·0001 |
| **Alanine aminotransferase, median (IQR), U/L** | 22 (15-25) | 26 (17-46) |  | 26 (17-46) | 29 (21-41) | 0·46 |
| > 50 U/L, No./total (%) | 1/10 (10·0) | 31/136 (22·8) |  | 25/116 (21·6) | 6/20 (30·0) | 0·59 |
| **Aspartate aminotransferase, median (IQR), U/L** | 24 (22-30) | 26 (20-35) |  | 26 (20-36) | 25 (21-34) | 0·64 |
| > 40 U/L, No./total (%) | 1/8 (12·5) | 24/108 (22·2) |  | 19/91 (20·9) | 5/17 (29·4) | 0·65 |
| **Total bilirubin, median (IQR), μmol/L** | 9·7 (6·0-18·0) | 10·5(7·0-16·9) |  | 10·8 (7·0-16·5) | 8·7 (7·0-18·3) | 0·94 |
| > 28 μmol/L, No./total (%) | 1/12 (8·3) | 14/135 (10·4) |  | 12/116 (10·3) | 2/19 (10·5) | > 0·99 |
| **Direct bilirubin, median (IQR), μmol/L** | 3·9 (2·8-7·3) | 4·0 (2·7-5·8) |  | 3·9 (2·6-5·4) | 4·0 (3·0-6·7) | 0·30 |
| > 10μmol/L, No./total (%) | 2/12 (16·7) | 11/135 (8·2) |  | 8/116 (6·9) | 3/19 (15·8) | 0·39 |
| **Serum creatinine, median (IQR), μmol/L** | 80·5 (69·3-89·3) | 74·3 (59·7-83·5) |  | 72·5 (59·0-83·5) | 76·2 (63·8-83·5) | 0·57 |
| > 106 μmol/L, No./total (%) | 1/12 (8·3) | 9/132 (6·8) |  | 7/112 (6·3) | 2/20 (10·0) | 0·90 |
| **Troponin T, median (IQR),** **pg/mL** | 3·00 (1·10-47·12) | 3·00 (0·03-9·7) |  | 3·00 (0·03-9·15) | 6·13 (0·23-13·40) | 0·23 |
| > 14 pg/mL, No./total (%) | 3/7 (42·9) | 8/80 (10·0) |  | 5/67 (7·5) | 3/13 (23·1) | 0·23 |
| **Procalcitonin, median (IQR), ng/mL** | 0·04 (0·03-0·21) | 0·05 (0·03-0·06) |  | 0·05 (0·03-0·06) | 0·05 (0·02-0·08) | 0·56 |
| > 0·05 ng/mL, No·/total (%) | 4/13 (30·8) | 61/128 (47·7) |  | 50/107 (46·7) | 11/21 (52·4) | 0·81 |
| **Erythrocyte sedimentation rate, median (IQR), mm/h** | 27 (21-51) | 22 (10-36) |  | 21(10-33) | 23 (7-44) | 0·77 |
| > 15 mm/h, No./total (%) | 10/11 (90·9) | 72/101 (71·3) |  | 61/88 (69·3) | 11/13 (84·6) | 0·42 |
| **C-reactive protein, median (IQR), mg/L** | 7·6 (0·9-31·8) | 7·2 (2·0-24·2) |  | 5·7 (1·9-15·6) | 31·7 (14·2-54·2) | < 0·0001 |
| > 5 mg/L, No./total (%) | 8/12 (66·7) | 66/122 (54·1) |  | 48/102 (47·1) | 18/20 (90·0) | 0·0010 |
| **CKMB**† |  |  |  |  |  |  |
| Increase at first test, No./total (%) | 2/11 (18·2) | 7/91 (7·7) |  | 6/79 (7·6) | 1/12 (8·3) | *P* > 0·99^*^ |

Abbreviations: IQR, Interquartile range; CKMB, Creatine Kinase-MB.

^*^ The *P*-value was derived from Fisher’s exact test, two-sided; ^#^ P-value for the comparison between severe cases versus severe infected patients.

† Classified by different reference range of hospitals.
